# Temporal and micro-spatial heterogeneity in the distribution of *Anopheles* vectors of malaria along the Kenyan coast

**DOI:** 10.1186/1756-3305-6-311

**Published:** 2013-10-28

**Authors:** Martin Walker, Peter Winskill, María-Gloria Basáñez, Joseph M Mwangangi, Charles Mbogo, John C Beier, Janet T Midega

**Affiliations:** 1 Department of Infectious Disease Epidemiology, School of Public Health, Faculty of Medicine (St Mary’s campus), Imperial College London, Norfolk Place, London W2 1PG, UK; 2 MRC Centre for Outbreak Analysis & Modelling, Department of Infectious Disease Epidemiology, Imperial College London, London W2 1PG, UK; 3 Vector Biology Department, KEMRI/Wellcome Trust Research Programme, P.O. Box 230–80108, Kilifi, Kenya; 4 Department of Public Health Sciences, Miller School of Medicine University of Miami, Miami, USA; 5 Department of Life Sciences, Division of Cell & Molecular Biology, Imperial College, South Kensington Campus, London SW7 2AZ, UK

**Keywords:** *Anopheles gambiae*, *Anopheles funestus*, Polynomial distributed lag generalized linear mixed models, Rainfall, Temperature, Household occupancy, Larval habitats, Mosquito density, Malaria, Kilifi, Kenya

## Abstract

**Background:**

The distribution of anopheline mosquitoes is determined by temporally dynamic environmental and human-associated variables, operating over a range of spatial scales. Macro-spatial short-term trends are driven predominantly by prior (lagged) seasonal changes in climate, which regulate the abundance of suitable aquatic larval habitats. Micro-spatial distribution is determined by the location of these habitats, proximity and abundance of available human bloodmeals and prevailing micro-climatic conditions. The challenge of analysing—in a single coherent statistical framework—the lagged and distributed effect of seasonal climate changes simultaneously with the effects of an underlying hierarchy of spatial factors has hitherto not been addressed.

**Methods:**

Data on *Anopheles gambiae* sensu stricto and *A. funestus* collected from households in Kilifi district, Kenya, were analysed using polynomial distributed lag generalized linear mixed models (PDL GLMMs).

**Results:**

Anopheline density was positively and significantly associated with amount of rainfall between 4 to 47 days, negatively and significantly associated with maximum daily temperature between 5 and 35 days, and positively and significantly associated with maximum daily temperature between 29 and 48 days in the past (depending on *Anopheles* species). Multiple-occupancy households harboured greater mosquito numbers than single-occupancy households. A significant degree of mosquito clustering within households was identified.

**Conclusions:**

The PDL GLMMs developed here represent a generalizable framework for analysing hierarchically-structured data in combination with explanatory variables which elicit lagged effects. The framework is a valuable tool for facilitating detailed understanding of determinants of the spatio-temporal distribution of *Anopheles*. Such understanding facilitates delivery of targeted, cost-effective and, in certain circumstances, preventative antivectorial interventions against malaria.

## Background

The density of *Anopheles* mosquito vectors relative to human hosts (the vector-to-host ratio) is a critical component of the intensity of malaria transmission [1]. Spatial and temporal heterogeneity in the density and distribution of anopheline mosquitoes is determined principally by the availability of aquatic habitats suitable for the maturation and development of their larvae [2,3]. Climate is a major determinant of macro-spatial (district, regional or country) and temporal heterogeneity in the distribution of such habitats and consequently of heterogeneity in the distribution of mosquito vectors [4-8]. In turn, climatic variation drives heterogeneity in the intensity of malaria transmission within and among human populations, both in the short- (seasonal) [9] and long- (climate change) terms [10]. The fine, micro-spatial distribution of vectors within communities depends on the location of aquatic habitats [11,12], the proximity of human bloodmeals [13,14] (*Anopheles gambiae* sensu stricto and *A. funestus* mosquitoes are strongly anthropophagic [15]) and micro-climatic conditions, particularly the direction of the prevailing wind [16] that transports olfactory cues from humans to female mosquitoes [17,18].

There is great interest in identifying factors underlying the macro- and micro-spatial and temporal distribution of anopheline vectors either mechanistically using mathematical models (e.g. [19-21]), or phenomenologically using statistical models (e.g. [22-24]). Studies are often motivated by projecting the effects of climate change on the distribution of anophelines [25-27] and endemic malaria [28]; improving targeted control on macro- [29,30] and micro-spatial [16,31] scales, or developing models with capacity to offer reliable predictions of impending malaria epidemics [32]. The latter ‘early warning’ prediction models are underpinned by the lagged relationship between meteorological variables, vector density and malaria incidence [33]. For example, depending on temperature, it takes between 6 and 47 days for mosquito larvae to develop into adults; between 4 and 111 days for the completion of sporogony within a mosquito following ingestion of an infected bloodmeal (infected mosquitoes are not infectious before completion of sporogony), and a further 10- to 16-day incubation period before an infected human develops symptoms of malaria (see Table 1 in [34]). Thus, increased anopheline densities [35,36], and cases of malaria [34], are associated with prior increases in rainfall [37].

**Table 1:** Descriptive statistics of the three sampled study villages in Kilifi district, Kenya

**Village**	**Number of samples/households**	**Mean**^**a**^**/range visits per household**	**Mean**^**a**^**/range distance from nearest mosquito larval habitat (m)**	**Mean**^**a**^**/range household occupancy**	**Total/mean**^**a**^**/variance**^**a **^**number of female *Anopheles *** **collected**	
					***A. gambiae***	***A. funestus***
Jaribuni	786/12	66/4–95	350/52–620	3.1/1–9	2,422/3.1/58	5,199/6.6/380
Majajani	948/23	41/1–86	220/52–630	2.9/1–9	1,911/2.0/52	495/0.52/11
Mtepeni	926/19	49/1–96	170/40–540	2.8/1–12	1,187/1.3/14	411/0.44/2.5

^a^Arithmentic mean (or variance) rounded to 2 significant figures. Mean values are per household.

Despite an extensive literature, there remains a lack of general consensus on the relative importance and predictive capacity of different meteorological factors [35]. This is partly due to the biological and ecological complexities that underpin the associations. For example, the association between increased rainfall and increased vector abundance would appear ostensibly simple, being mediated by more numerous and larger aquatic habitats. However, the persistence of such habitats following rain also depends on rates of evaporation, which are themselves driven by a myriad of factors, including temperature, atmospheric pressure, wind-speed, humidity and the surface area of the specific habitat [19]. Furthermore, temperature-dependent rates of larval development [38-40] ensure that the lag between increased rainfall and increased abundance of mosquitoes will vary with temperature; at an average temperature of 16°C larvae become adults in an average of 47 days, while at 30°C it takes an average of 10 days (see Table 1 in [34] and Figure 1 in [41]). Moreover, when inference is made using data on malaria incidence, temperature-dependent rates of sporogony and the incubation period in the human, add an additional and uncertain lag time.

**Figure 1:**
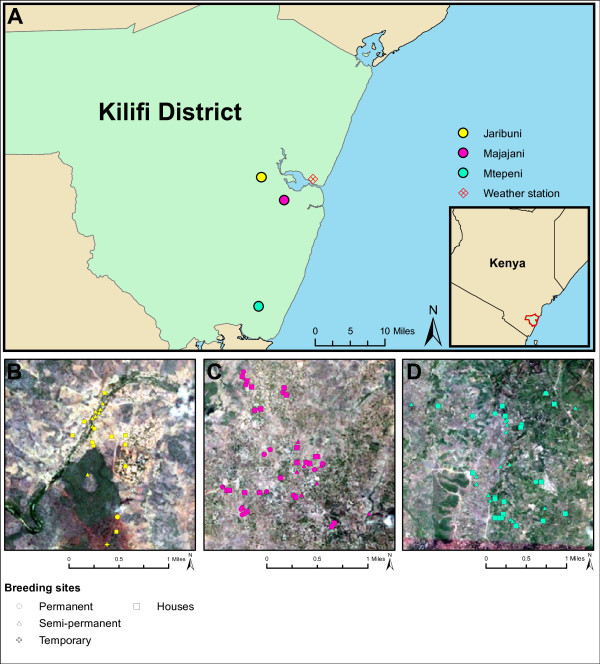
**Locations of the studied communities, households and anopheline larval habitats.** Panel **A** depicts the locations of the studied communities of Jaribuni, Majajani and Mtepeni within Kilifi district, which is located in south-east Kenya (inset). The location of the national weather station (within the town of Kilifi) from which the meteorological data were collected is also indicted (grid marker). Panels **B**, **C** and **D** are, respectively, satellite images of Jaribuni (yellow markers), Majajani (pink markers) and Mtepeni (green markers) overlaid with the locations of the sampled households (square markers) and anopheline larval habitats. Larval habitats are classified as permanent (e.g. riverbanks, large ponds and swamps; circles); semi-permanent (e.g. streams, pools, and small ponds; triangles), or temporary (e.g. small streams, and small pools created by tire tracks and damaged infrastructure; crosses).

Methodological issues related to the analysis of these complex associations are also likely to contribute to somewhat conflicting conclusions on the relative importance of different meteorological variables. The most common analytical approach has been to calculate pairwise correlations between malaria incidence and variables such as daily rainfall (e.g. [42]), temperature (e.g. [43]) and humidity (e.g. [44]) at different numbers of days in the past (lags). This univariate approach is not conducive to multivariate adaptation, making it prone to confounding by inevitably (highly) correlated meteorological variables. Furthermore, the method does not quantify the overall statistical significance nor the explanatory power of variables considered at different lags.

Polynomial distributed lag (PDL) models [45] provide a solution to these analytical difficulties, permitting the association of multiple explanatory (meteorological) variables over a continuum of lags to be estimated as part of a single, coherent model structure. Such models have been used previously to estimate the association of rainfall, and daily temperature with weekly incidence of malaria in Ethiopia distributed over a 10-week lag period [34]. The PDL framework can be applied to explanatory variables in a generalized linear model (GLM) [46]. The resulting PDL GLM is adaptable to hierarchical data structures, becoming a polynomial distributed lag generalized linear mixed model (PDL GLMM, Table 2) [47,48]. Such frameworks permit modelling of explanatory variables acting on a variety of scales; from climatic (meteorological) data acting at the macro-spatial scale, to temporally-dependent factors acting below the level of the (longitudinally sampled) units of observation. This potential of the PDL GLMM framework has not been applied to the analysis of data on vector abundance, and consequently little is known about the relative importance and predictive capacity of variables acting on multiple hierarchical scales.

**Table 2:** Polynomial distributed lag generalized linear mixed models (PDL GLMMs)

		
**Generalized linear mixed models (GLMMs)**		**Polynomial distributed lag models (PDLMs)**
Generalized linear models (GLMs) extend general linear regression models to the analysis of data with non-normally distributed error structures arising from the exponential family of probability distributions. Discrete (count) distributions within this family include the binomial, Poisson, and negative binomial distribution with *known* overdispersion parameter *k*. Generalized linear mixed models (GLMMs) are GLMs that include both fixed and random effects. Fixed effects are represented by a measured explanatory variable (covariate) and are quantified by regression coefficients. By contrast, random effects embody the unmeasured or unmeasurable characteristics of a unit of observation which induce correlation (clustering) among data collected from the same unit; e.g. numbers of mosquitoes collected from the same household. Random effects are quantified in terms of variability (variance) among data collected from distinct units of observation.		Polynomial distributed lag models (PDLMs) are suitable for analysing data where one or more explanatory variables exert a lagged effect on the collected (response) data; e.g. rainfall at some point in the past affects mosquito abundance now. Moreover, PDLMs assume that this effect is *distributed* over the entire lag period; e.g. rainfall over the past several weeks affects mosquito abundance now. Treating every point in the past as a separate explanatory variable with its own unique coefficient becomes infeasible for all but very short lag periods; it is impractical to estimate a large number of coefficients of often highly correlated explanatory variables (e.g. rainfall yesterday is correlated with rainfall today). This problem is avoided by PDLMs using a polynomial functional form with ample flexibility to capture the shape of the distributed effect.

In this paper, mixed (containing random effects) PDL models (Table 2) are used to analyse longitudinal data on the abundance of *Anopheles gambiae* and *A. funestus*, collected between 2000 and 2002 from households located in Kilifi district, adjoining the Kenyan coast, an area endemic for falciparum malaria. Research on anopheline mosquitoes in Kilifi, ongoing since the early 1990s [23,49-54], has identified the importance of environmental heterogeneity in affecting the abundance and productivity of mosquito larval habitats [55], the distribution of adult mosquitoes, and the incidence of malaria due to *Plasmodium falciparum*[23,56], the latter having declined in recent years [57,58].

The PDL GLMM models presented here incorporate the (lagged) meteorological covariates of daily rainfall and maximum temperature, and the household-level covariates of distance from the nearest aquatic larval habitat and occupancy (number of people in the household) with the aim of: a) exemplifying the flexibility and power of the PDL GLMM framework to analyse longitudinal, hierarchical and overdispersed count data; b) identifying factors underlying the observed spatial and temporal patterns as a source of baseline information prior to the recent decline in malaria transmission and vector density in Kilifi [59], and c) providing information which may influence the design and application of targeted, cost-effective and preventative anti-vectorial interventions for malaria control.

## Methods

### Study area, household sampling, and ethical considerations

The study was conducted in the villages of Jaribuni (39°44′E 3°37′S) (site 1), Majajani (39°74′E 3°40′S) (site 2), and Mtepeni (39°45′E 3°53′S) (site 3) in Kilifi District, along the Kenyan coast over the period from May 2000 through April 2002 (Figure 1A). Random samples of approximately 30% of all households in each community were selected for potential inclusion in the study. Written informed consent was sought from the heads of households to permit mosquito collection after the study was explained in their local language. Households that did not consent did not participate further. The initial study design was to perform mosquito catches in each participating household fortnightly throughout the two-year study period. However, due to householders sometimes being absent and, more commonly, requesting additional catches, the mean number of catches performed per household varied between 41 and 66 (Table 1). Most households were visited and sampled on multiple occasions over the study, but not more than once per week. The maximum number of visits to a household was 96, the minimum was 1 (Table 1). The study was reviewed and approved by the Institutional Review Board of the Kenya Medical Research Institute (KEMRI), Nairobi, Kenya.

### Entomological data

*Anopheles* mosquitoes were sampled using the pyrethrum spray catch (PSC) method [60]. Mosquitoes from each household were held separately in labelled paper cups, stored in a cool box, and transported to the laboratory. In the laboratory, all mosquitoes were immediately transferred to a -20°C freezer for approximately 10 minutes. Each mosquito sample was allocated a unique identification number and separated by morphospecies into *A. gambiae* and *A. funestus* using the morphological criteria of Gillies and Coetzee [61]. The legs and wings of each *Anopheles* female were then detached from the rest of the body and stored dry on silica gel in labelled vials before being processed for molecular differentiation of the *A. gambiae* and *A. funestus* sibling species using the methods of Scott *et al.*[62] and Koekemoer *et al*. [63]. Only *A. gambiae sensu stricto* (*s.s.*) (henceforth referred to simply as *A. gambiae*) was identified among the *A. gambiae s.l.* samples (i.e., these contained no *A. arabiensis* or *A. merus*, the other members of the *gambiae* complex that have been reported along the Kenyan coast [23]). Likewise, *A. funestus* was found to comprise *A. funestus s.s*. and is henceforth referred to as *A. funestus*.

### Household covariates

All households participating in mosquito sampling were mapped using a hand held global positioning system (GPS) device (Garmin International Inc., Olathe, KS USA). Larval habitats within a 1 km distance from the study households were also mapped (Figures 1B, 1C, 1D). Samples of mosquito larvae were collected from each habitat and were morphologically identified as anophelines (as opposed to other culicines). Larvae were not disaggregated by *Anopheles* species (complexes). Distance from the larval habitats to the nearest household was calculated using R [64]. In addition, the number of individuals sleeping in the household the night before mosquito sampling was recorded on field data forms during mosquito sampling. Note that the number of individuals sleeping in a household changed over time and so was included at the observation-level (rather than the household-level) in the statistical model.

### Meteorological data

Daily meteorological data on the rainfall and minimum and maximum temperatures were obtained from the national weather station located at the Kilifi Institute of Agriculture (3°37′15″S 39°50′45″E) within the town of Kilifi (Figure 1A). The weather station lies 10.8, 11.5 and 34.1 km to the north east of Jaribuni, Majajani and Mtepeni respectively. This weather station serves the whole of Kilifi district and represents the most accurate source of data available on the climate in the three study communities. For the fewer than 1% of days that data were missing, values were imputed by linearly interpolating to the midpoint between the preceding and following days for which data were recorded. There were no instances of missing data on more than one consecutive day. It was assumed that the meteorological data represent accurately the climatic conditions in the surrounding study sites.

### Statistical framework

Data on the number of *A. gambiae* or *A. funestus* were modelled separately using a generalized linear mixed model (GLMM). Let *Y*_*ij*_ denote observation *j* (number of mosquitoes) collected from household *i*. It was assumed that *Y*_*ij*_ was Poisson distributed with mean and variance, *μ*_*ij*_, given by the following log-linear mixed effects model,

(1) $$\begin{array}{l} \ln\;\mu_{\mathrm{ij}}=\alpha x_{\mathrm{ij}}+\beta z_{i}+\epsilon_{i}+e_{\mathrm{ij}},\\ \epsilon_{i}\sim N\left(0,\delta_{H}\right),\\ e_{\mathrm{ij}}\sim N\left(0,\delta_{O}\right). \end{array}$$

Here **x**_*ij*_ and **z**_*i*_ are observation-level and household-level vectors of covariates respectively. The covariate vector **x**_*ij*_ comprises the number of people sleeping in the household the night before day *t*_*ij*_ after the start of the study, and the daily rainfall and temperature lagged from *p*_min_ to *p*_max_ days into the past. For example, if *p*_min_ = 5 and *p*_max_ = 10, **x**_*ij*_ would include the daily rainfall and temperatures 5–10 days prior to day *t*_*ij*_. Day *t*_*ij*_ is also included as a continuous covariate in **x**_*ij*_. The inclusion of *t*_*ij*_ accounts for any systematic (non-seasonal) linear changes in the density of the mosquito population over the study period. Furthermore, this relationship was permitted to vary among sites by including an appropriate interaction term. The household-level covariate vector, **z**_*i*_, comprises the distance of the household from the nearest mosquito larval habitat, and the location (site) of the household (Jaribuni, Majajani or Mtepeni). In the log-linear model the coefficients measure the multiplicative effect on the mean number of mosquitoes per household of the covariate in question.

The error term *ϵ*_*i*_ in Eqn. (1) is a household-level, normally distributed random effects term that accounts for the possibility that mosquitoes cluster within households. On the natural logarithmic scale, the magnitude of this clustering is quantified by the variance $\delta_{H}^{2}$. Converting to the scale of counts (as opposed to log counts), the clustering is quantified by $\sigma_{H}^{2}=\text{exp}\left(2\delta_{H}^{2}\right)–\text{exp}\left(\delta_{H}^{2}\right)$. Household clustering causes extra-Poisson variation (overdispersion) in the numbers of mosquitoes per household. It was particularly important to account for household clustering in this analysis due to the potential for heavily infested households to have been sampled somewhat disproportionately (some householders received by request additional unplanned mosquito catches, see Methods*, Study area, household sampling, and ethical considerations*). The residual overdispersion that is not due to household clustering is accounted for by the random effects term *e*_*ij*_ in Eqn. (1); this parameter is normally distributed, independent of *ϵ*_*i*_ and specific to each observation. That is, it is an observation-level random effect. On the natural logarithmic scale, the residual overdispersion is quantified by $\delta_{O}^{2}$. On the original scale of the counts, it is quantified by $\sigma_{O}^{2}=\text{exp}\left(2\delta_{O}^{2}\right)–\text{exp}\left(\delta_{O}^{2}\right)$. Modelling residual (within household) overdispersion using an observation-level random effect [65,66] has two principal advantages over alternative methods such as negative binomial or quasi-Poisson regression. First, the model is maintained as a GLMM, permitting likelihood-based fitting and coefficient estimation within a well-developed framework [47,48]. Second, overdispersion is modelled in a hierarchical manner (observations nested within households) permitting direct comparison of the relative contribution of random variation at observation and household levels (viz. comparison of $\sigma_{H}^{2}$ and $\sigma_{O}^{2}$).

### Polynomial distributed lag model

In the model described by Eqn. (1), the number of coefficients to estimate for a particular meteorological covariate is equal to the number of lags considered. For example, if daily rainfall and temperature were lagged from 5 to 15 days into the past (*p*_min_ = 5, *p*_max_ = 15), it would be necessary to estimate 10 coefficients for each meteorological covariate. Coefficients estimated using this so-called unconstrained lag structure are unstable because of collinearity among different lags of the same variable [45]. A solution to this is to constrain the coefficients by forcing them to take the shape of a function of the lag. The Almon lag model [67] uses an *n*^th^ degree polynomial as this functional form,

(2) $$\beta_{l}=\sum_{k=0}^{k=n}\theta_{k}l^{k}.$$

Here *β*_*l*_ represents the coefficient of a variable at lag *l*. That is, *β*_*l*_ is a coefficient of either rainfall, or maximum temperature *l* days in the past. Parameter *θ*_*k*_ is the *k*^th^ coefficient of the *n*^th^ degree polynomial. Thus, the number of estimated coefficients (the *θ*_*k*_s) associated with each lagged meteorological covariate is reduced from the number of lag days to the order of the polynomial, *n*, giving rise to the PDL.

### Model fitting

The model was fitted to the data using GLMM techniques [47,48] implemented using the lme4 package for R [64]. Following Teklehaimanot *et al.*[34], the polynomial describing the relationship between the number of lag days and the coefficients of rainfall and temperature was set to order 4 (*n* = 4). Preliminary analyses indicated that this order polynomial gave ample flexibility to capture variously shaped relationships. The range of lags considered was motivated by mosquito biology. The time from oviposition of eggs in an aquatic habitat to the development of an adult mosquito depends on temperature, varying between about 7 days (1 week) at 40°C to 47 days (approximately 7 weeks) at 16°C [34,68,69].

## Results

### Descriptive statistics

Table 1 gives descriptive statistics of the three study sites; the villages of Jaribuni, Majajani and Mtepeni in Kilifi district, Kenya (Figure 1). Majajani was the most sampled village; 948 mosquito catches were performed in 23 households. The least sampled village was Jaribuni, with 786 catches undertaken in 12 households. The density of *Anopheles* mosquitoes was highest in Jaribuni; the mean number of *A. gambiae* and *A. funestus* per household was 3.1 and 6.6 respectively. Overall, Table 1 indicates that there was considerable heterogeneity in the mean *Anopheles* densities among the three sites. In particular, the mean density of *A. funestus* was thirteen-fold and fifteen-fold larger in Jaribuni compared with Majajani and Mtepeni respectively. The distances of households from the nearest mosquito larval habitats were also heterogeneous among sites. For instance, the average distance of a household from a larval habitat in Mtepeni was approximately half that of the corresponding mean distance in Jaribuni. The average number of people sleeping in a household the night prior to mosquito sampling was broadly similar among sites (Table 1).

### Fitted model

**Figure 2:**
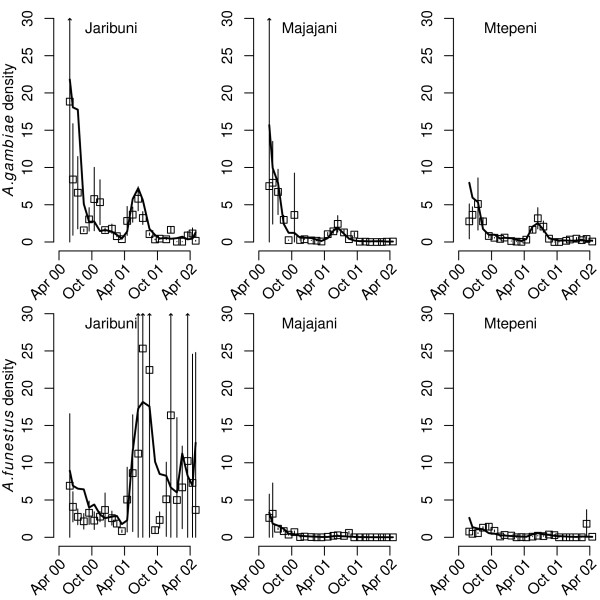
**Observed and model-predicted household densities of *Anopheles *mosquitoes.** Depicted in each panel are the observed and model-predicted mosquito densities (the number of mosquitoes per household) in the different sampled sites within Kilifi district; the villages of Jaribuni, Majajani and Mtepeni. Squares represent the observed data averaged (arithmetic means) by month, and the vertical lines indicate the respective 95% confidence intervals. The solid black lines indicate the mosquito densities predicted from the fitted statistical model. Note that the statistical model was fitted separately to species-specific mosquito counts.

Figure 2 depicts the observed and model-predicted mean *Anopheles* densities per household in the three study sites. In general, the model fits the observed *A. gambiae* data well; the model-predicted mean falls within the 95% confidence intervals of the majority of the observed data across all sites. Similarly, the model also fits well to the observed *A. funestus* data from Majajani and Mtepeni. The least good fit is to the data on the household densities of *A. funestus* from Jaribuni, where the mean density was particularly high compared with Majajani and Mtepeni (Table 1).

### Meteorological covariates

**Figure 3:**
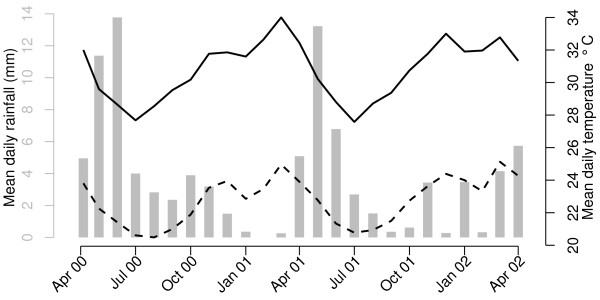
**Mean daily rainfall and temperature between April 2000 through April 2002 in Kilifi district, Kenya.** The x-axis indicates time from 7 weeks before the start of the study period through to the end in accordance with the maximum and minimum lag periods considered in the statistical model (see main text). The grey bars indicate the mean daily rainfall whose scale is given by the grey y-axis on the left hand side. The black solid and dashed lines indicate, respectively, the maximum and minimum mean daily temperatures whose scale is given by the black y-axis on the right hand side.

Rainfall and maximum temperature from 7 weeks before the start of the study period, in April 2000, to its end in April 2002 are depicted in Figure 3. This timeframe is in accordance with the 7-week range of lags considered for these meteorological covariates in the statistical model. The data indicate two pronounced rainy seasons, peaking around May or June. Temperatures tend to be highest between, approximately, November and May, and lowest between, approximately, June and October. It is also clear from Figure 3 that minimum and maximum daily temperature are strongly positively correlated (Pearson’s correlation coefficient = 0.77). This produced problems of multicollinearity [70] when both variables were included in the statistical model. Preliminary analysis indicated that maximum daily temperature was a better predictor of mosquito density than minimum daily temperature and so only the latter was included in the final model.

The strength and statistical significance of the association between the meteorological data (daily rainfall and maximum temperature) and *Anopheles* density is shown by the results of an analysis of deviance given in Table 3. These results indicate that daily rainfall and maximum temperature are strongly and statistically significantly associated with the density of *A. gambiae* and *A. funestus.*

**Table 3:** **Analysis of deviance of covariates associated with the household density of *Anopheles *** **mosquitoes**

**Covariate**	**DF**^**a**^	**Species**^**b**^	**∆Dev**^**c,d**^	***P-*** **value**^**d,e**^
Rainfall^f^	5	*A. gambiae*	80	< 0.001
		*A. funestus*	24	< 0.001
Maximum temperature^f^	5	*A. gambiae*	57	< 0.001
		*A. funestus*	27	< 0.001
Days since study start date × village	5	*A. gambiae*	120	< 0.001
		*A. funestus*	180	< 0.001
Household occupancy the night before mosquito collection	5	*A. gambiae*	8.9	0.11
		*A. funestus*	31	< 0.001
Distance of household from nearest mosquito larval habitat	6	*A. gambiae*	5.2	0.51
		*A. funestus*	12	0.070

^a^Degrees of freedom.

^b^Models fitted separately to species-specific counts per household.

^c^Difference between the (residual) deviance of the model with all covariates and the model without the covariate in question [46].

^d^Rounded to 2 significant figures.

^e^Calculated assuming that, under the null hypothesis (of no statistically significant association between the covariate and the numbers of mosquitoes per household), ∆Dev is *χ*^2^ distributed with the indicated degrees of freedom [71].

^f^Included as a 4th order polynomial distributed lag term yielding 5 degrees of freedom.

**Figure 4:**
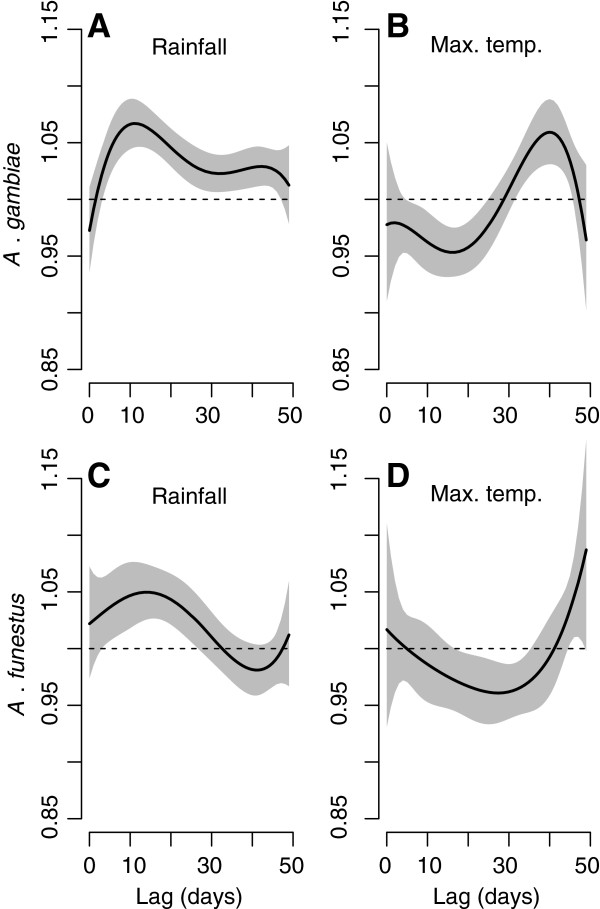
**Distributed lag structure of the association between daily rainfall and maximum temperature on the density of *Anopheles *mosquitoes.** The solid lines in each panel represent the model-predicted multiplicative effect on the mean number of mosquitoes per household (mosquito density) associated with either a 1 mm increase in daily rainfall (Panels **A** and **C** for *A. gambiae* and *A. funestus* respectively), or a 1°C increase in daily temperature at each lag time (Panels **B** and **D** for *A. gambiae* and *A. funestus* respectively). The grey shaded areas indicate 95% confidence intervals. The structure of the relationship between the coefficient and the lag time is constrained by a 4th order polynomial.

The structure of the association between the meteorological covariates and mosquito density over the 7-week lag is depicted in Figure 4. A statistically significant association is suggested at lags (days in the past) where the confidence interval for the multiplicative effect on mean mosquito density does not include 1. Bearing this in mind, rainfall between 4 and 47 days lag is positively (an increase in rainfall leads to an increase in mosquito density) and statistically significantly associated with the density of *A. gambiae* (4A). For the density of *A. funestus*, the association is also positive and statistically significant between 4 and 27 days lag (4C). Maximum temperature is negatively and statistically significantly associated with the density of *A. gambiae* between 5 and 24 days lag (4B), and with *A. funestus* density between 17 and 35 days lag (4D). A statistically significant positive association between maximum temperature and *A. gambiae* density occurs between 29 and 47 days in the past, and marginally between 45 and 48 days for the density of *A. funestus*.

### Household covariates

**Figure 5:**
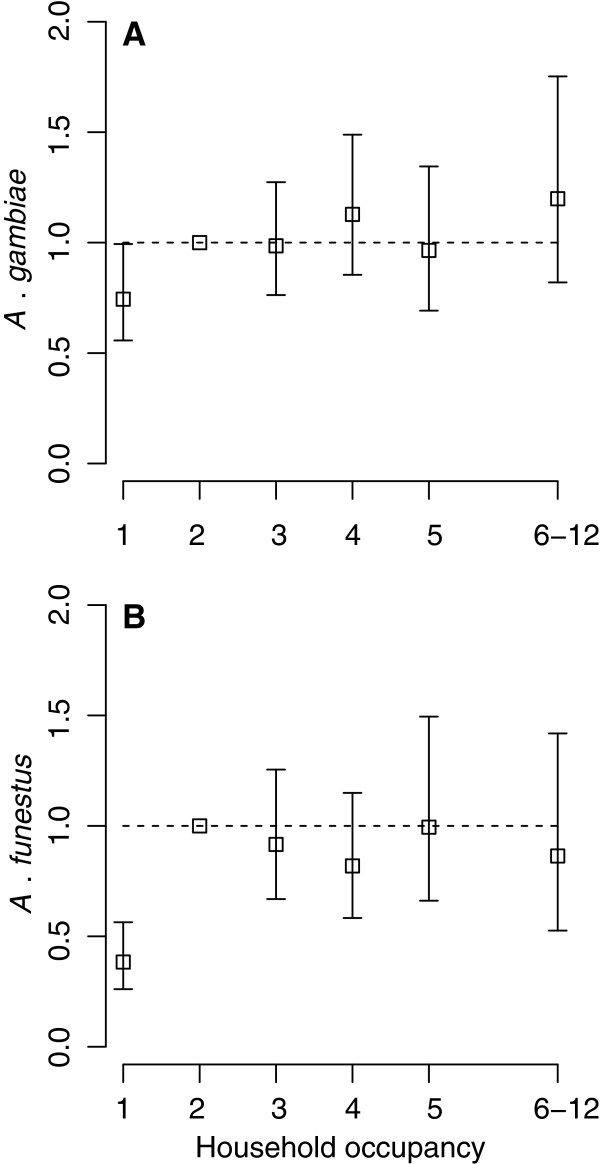
**The association between mosquito density and household occupancy the night before mosquito collection.** The squares in each panel (**A** for *A. gambiae* and **B** for *A. funestus*) represent the model-predicted multiplicative effect on the mean number of mosquitoes per household (mosquito density) associated with household occupancy (the numbers of people sleeping in the household during the night prior to sampling). Error bars indicate 95% confidence intervals. Note that for a household occupancy of 6–12 the square is plotted at the mean of (6.7) within this category.

The results in Table 3 indicate that the number of people sleeping in the household during the night prior to sampling—hereafter referred to as household occupancy—is strongly and statistically significantly associated with the density of *A. funestus* but not significantly associated with the density of *A. gambiae.* Figure 5 shows that the density of *A funestus* increases sharply from household occupancies of 1 to 2, thereafter remaining relatively constant. The relationship between household occupancy and the density of *A gambiae* is more linear (and positive), albeit not statistically significant (Table 3 and Figure 5A).

**Figure 6:**
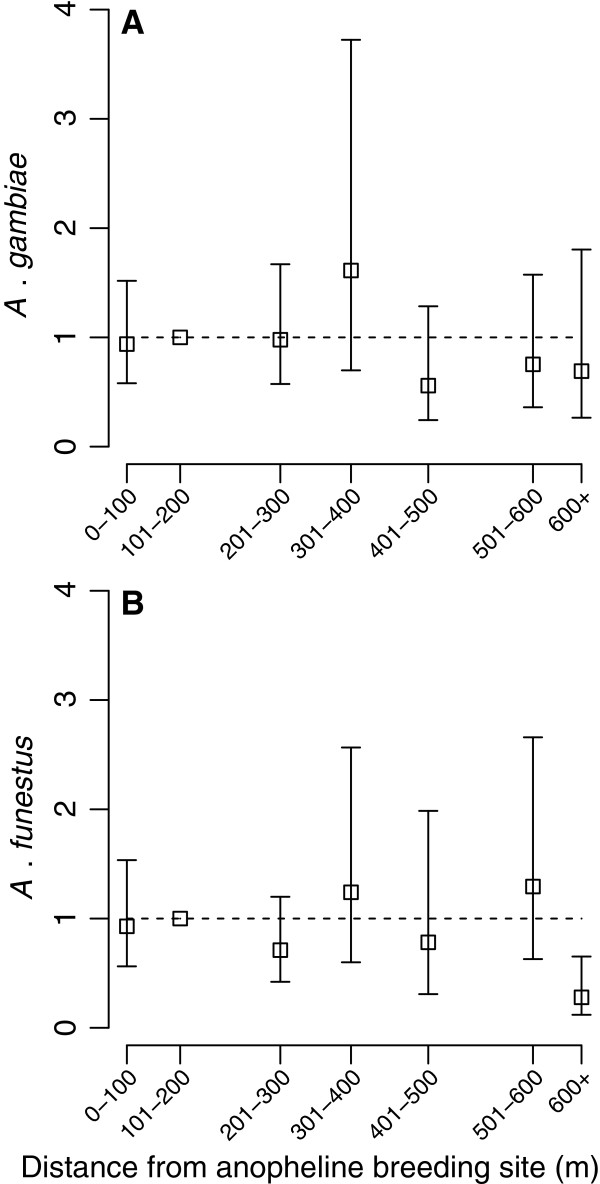
**The association between mosquito density and the distance of a household from the nearest mosquito larval habitat.** The squares in each panel (**A** for *A. gambiae* and **B** for *A. funestus*) represent the model-predicted multiplicative effect on the mean number of mosquitoes per household (mosquito density) associated with the distance of a household from the nearest mosquito larval habitat. Error bars indicate 95% confidence intervals. Squares are plotted at the mean distance within each category.

Overall, the distance of the household from the nearest mosquito larval habitat is not a statistically significantly predictor of the density of either *Anopheles* species (Table 3 and Figure 6). However, the density of *A. funestus* in households situated over 600 metres from the nearest larval habitat is statistically significantly lower (*P*-value = 0.0032) than that in households located in the baseline distance category of 101–200 metres for the nearest larval habitat (Figure 6B).

### Household clustering and overdispersion

The total amount of extra-Poisson variation (overdispersion) in the observed numbers of mosquitoes per household is quantified by the sum of the variance terms, $\sigma_{H}^{2}$ and $\sigma_{O}^{2}$. Parameter $\sigma_{H}^{2}$ quantifies the amount of overdispersion induced by the clustering of mosquitoes within households. Parameter $\sigma_{O}^{2}$ quantifies the residual overdispersion having accounted for that induced by household clustering. Thus, from the estimates of $\sigma_{H}^{2}$ and $\sigma_{O}^{2}$ given in Table 4, it can be seen that 0.55% [0.4/(72 + 0.4)] and 0.06% [0.22/(380 + 0.22)] of the total overdispersion in, respectively, *A gambiae* and *A. funestus* numbers per household was accounted for by household clustering. Despite this low contribution to the overall degree of overdispersion, household clustering for both *Anopheles* species is statistically significant (Table 4).

**Table 4:** **Likelihood ratio tests of household clustering and overdispersion in household densities of *Anopheles *** **mosquitoes**

**Parameter**	**DF**^**a**^	**Species**	**Estimate**^**b**^	**LRS**^**b,c**^	***P-*** **value**^**d**^
Household clustering, $\sigma_{H}^{2}$	1	*A. gambiae*	0.40	51	< 0.001
		*A. funestus*	0.22	18	< 0.001
Overdispersion, $\sigma_{O}^{2}$	1	*A. gambiae*	72	6300	< 0.001
		*A. funestus*	380	8100	< 0.001

^a^Degrees of freedom.

^b^Rounded to 2 significant figures.

^c^Likelihood ratio statistic.

^d^Calculated assuming that, under the null hypothesis (of the parameter in question not statistically significantly improving the log-likelihood of the fitted model), the LRS is *χ*^2^ distributed with the indicated degrees of freedom [71].

## Discussion

The mixed PDL models, or PDL GLMMs (Table 2), applied in this analysis have been used to elucidate factors influencing micro-spatial and temporal heterogeneity in the distribution of anopheline vectors of malaria caught within households in Kilifi district, Kenya. Temporal variation in the abundance of *A. gambiae* and *A. funestus* is driven by seasonal changes in climate, specifically the amount of rainfall from 4 to 47 days in the past (depending on *Anopheles* species), and maximum daily temperature. The PDL component of the statistical framework has enabled the distributed shape of these relationships to be elucidated for the first time. Micro-spatial variation is related to the distance of households from the nearest anopheline larval habitat, and whether such households have single- or multiple-occupancy by people pernoctating in them on the night prior to sampling. Despite accounting for these explanatory variables, the abundance of mosquitoes is highly overdispersed and clustered among households, effects which were quantified by the GLMM component of the modelling framework.

Strong empirical associations between anopheline abundance and seasonal variations in rainfall and temperature have been demonstrated previously [5,23,72], as have differences in these associations among species [73], although not using a PDL GLMM approach*.* The PDL models used here permitted a detailed description of the temporally distributed (lagged) nature of these associations which have not been previously elucidated (although the distributed lagged relationship between meteorological variables and cases of clinical malaria have been investigated in a similar fashion [34]). For example, for *A. gambiae*, rainfall was positively associated with mosquito abundance between 4 and 47 days in the past, with a peak in magnitude at approximately 11 days, and for *A. funestus*, between 4 and 27 days, with a peak at approximately 14 days. Rainfall acts principally to create and maintain viable larval habitats [74], but the exact shapes of these associations (Figure 4A for *A. gambiae*, Figure 4C for *A. funestus*) are likely to be the products of a multitude of interacting factors such as the local topology, cumulative effects of rainfall and heterogeneity in mosquito biology, the type, size and shape of larval habitats, in addition to other meteorological variables such as temperature, humidity and evaporation rates.

The distributed association of anopheline abundance with maximum daily temperature is more complex (Figure 4B for *A. gambiae*, Figure 4D for *A. funestus*); a negative association is indicated at short to medium lags—as observed previously in this area [72]—while a positive association arises at longer lag times. The effect of temperature on mosquito abundance is well documented for the extremes of the species tolerance levels [75,76]. This study sits within these extremes, where the mechanisms by which lagged temperatures may affect mosquito abundance are many and more nuanced, complicating interpretation of statistical (phenomenological) associations. High temperatures may reduce the size and abundance of larval habitats, not only affecting opportunities for breeding, but also altering local ecology, affecting regulatory factors such as the severity of density-dependent competition, the concentration of resources, and water quality among others. Furthermore, temperature influences the development time of anopheline aquatic stages [38-40], possibly interacting with the length of lags for other meteorological variables, such as rainfall. Temperature may also affect adult survival [77,78] and behaviour [79], the latter potentially altering the chance of an individual mosquito being trapped.

Among the micro-spatial determinants of household anopheline abundance, distance to the nearest suitable larval habitat was not, overall, statistically significantly associated with either species (although the density of *A. funestus* was statistically significantly lower for households located over 600 m from a larval habitat compared with households situated closer to larval habits, see Figure 6). Previous studies have shown that distance to larval habitat is negatively associated with anopheline density [80,81]. The lack of association in this analysis probably reflects, at least in part, a limitation of the GPS data, which were only recorded to an accuracy of approximately 31 metres. A further limitation is that the statistical framework does not include explicit spatial structure. That is, the distribution of larval habitats around individual households could not be considered. This led to the implicit modelling assumption that the dominant source of anophelines was the larval habitat nearest to a household. In principle, the PDL GLMM framework could be incorporated into spatial statistical models. This would permit more powerful interrogation of geo-referenced data and improve the capacity of existing spatial frameworks to capture accurately the effects of meteorological variables.

The intensity of human-derived sensory cues, such as carbon dioxide [82], probably underlies the association between the number of household inhabitants the night prior to mosquito sampling (household occupancy) and the number of anophelines caught; more inhabitants generate more intense cues, attracting more mosquitoes. However, the observed strength of this association may be diluted somewhat because houses inhabited by more people tended to be larger (although most houses comprise 2–3 rooms only), catches were made in a single room, and mosquitoes presumably distribute, at least to a certain extent, throughout the house. Moreover, the association may be confounded by the number of occupants in nearby households, which serve to make clusters of high-occupancy households more attractive than low-occupancy household clusters [31]. These factors may explain the lack of a statistically significant association with *A. gambiae*, a result which is surprising in the context of the highly anthropophilic, anthropophagic and endophagous nature of this species. A lack of association has been observed between the number of inhabitants in a house and malaria prevalence in the region [83].

The species-specific heterogeneity in abundance among the villages is consistent with previous studies in this area [72]. In particular, the markedly greater abundance of *A. funestus* in Jaribuni is attributable to local ecological heterogeneities [23]. Jaribuni lies next to a permanent river (Figure 1B), which provides plentiful and persistent larval habitats. Such habitats are more favourable to *A. funestus* which tolerate larger, more permanent larval habitats, whereas *A. gambiae* prefers more localised and transient fresh water habitats [2,84].

Identifying factors driving micro-spatial and temporal heterogeneity in the distribution of anopheline mosquitoes facilitates understanding of the causal pathway which drives the coupled dynamics of malaria transmission. In this regard, this study would have benefited from collecting additional public-health oriented outcome data. For example, measurement of standard entomological indices such as the entomological inoculation rate (EIR) during the study period would have improved our understanding of how the results relate to the incidence of falciparum malaria in the region. Nevertheless, in the context of malaria vector control, understanding spatial and temporal determinants of anopheline abundance can assist with judicious identification of high-risk communities [8,85], or even households, improving the cost-effectiveness of targeted interventions [81,86]. Moreover, the lagged nature of meteorological predictors of anopheline abundance can be exploited to predict when malaria outbreaks are likely to occur [32], guiding pre-emptive interventions.

Malaria epidemiology in sub-Saharan Africa has undergone significant changes in recent years [87]. Kilifi, like many areas, has seen a large increase in the distribution of insecticide-treated bednets (ITNs) since these entomological surveys were undertaken [88,89]. In other areas changes in bednet use has been associated with changes in entomological ecology, such as declines in *A. gambiae s.s.*[90]. The influence of the changing intervention landscape on *A. funestus* is less well understood. This study therefore provides an important baseline assessment of the entomological situation prior to scaling up of ITN distribution and provides a valuable comparative source for ongoing studies in the region.

## Conclusions

The statistical framework applied in this analysis, created by bringing together PDL models and GLMMs, represents a powerful tool for understanding factors underpinning the temporal and spatial distribution of anopheline mosquitoes, vectors of falciparum malaria in the study area. The PDL component of the framework permits modelling of past (lagged) seasonal changes in climate, which affects current mosquito density by mediating the abundance of aquatic larval habitats on a large, macro-spatial scale. The GLMM component permits simultaneous incorporation of finer, micro-spatial determinants of mosquito density while handling robustly any clustering of mosquito counts within units of observation (here, households) and residual overdispersion (extra-Poisson variation).

Continued development and application of appropriate and powerful statistical methods to understand better the determinants of anopheline abundance and malaria incidence will facilitate refinement of public health interventions. Detailed understanding of the impact of spatially and temporally heterogeneous explanatory variables will help to identify and target communities most at risk of malaria, improving the efficiency and cost-effectiveness of control efforts. In particular, a better understanding of the lagged effect of seasonal changes in climate on vector abundance will improve temporal targeting of vector control and other interventions. In turn, including this type of information into early warning systems may assist in the deployment of preventative anti-vectorial measures for malaria control, facilitating better allocation of resources in often resource-poor settings.

## Abbreviations

DF: Degrees of freedom; EIR: Entomological inoculation rate; GLMM: Generalized linear mixed model; GPS: Global positioning system; ITN: Insecticide-treated net; KEMRI: Kenya Medical Research Institute; PDL: Polynomial distributed lag; LRS: Likelihood ratio statistic; PSC: Pyrethrum spray cath; s.l.: Sensu lato; s.s.: Sensu stricto.

## Competing interests

The authors declare that they have no competing interests.

## Authors’ contributions

JTM and JMM coordinated the field work, data collection and data curation. JCB and CM provided overall project supervision and scientific guidance. MW, PW and MGB conceived the analytical approach. PW performed preliminary statistical analysis of the data. MW performed the final analysis. MW, PW, MGB and JTM wrote the manuscript. All authors read and approved the final, submitted version.
